# Design and Characterization of a DNA Vaccine Based on Spike with Consensus Nucleotide Sequence against Infectious Bronchitis Virus

**DOI:** 10.3390/vaccines9010050

**Published:** 2021-01-14

**Authors:** Lei Zuo, Wenjun Yan, Zhou Song, Hao Li, Xin Xie, Kui Gu, Peng Ma, Yiming Tian, Changyu Zhou, Yu Zhao, Xin Yang, Hongning Wang

**Affiliations:** Key Laboratory of Bio-Resource and Eco-Environment of Ministry of Education, Animal Disease Prevention and Food Safety Key Laboratory of Sichuan Province, College of Life Sciences, Sichuan University, Chengdu 610064, China; zuolei0104@foxmail.com (L.Z.); yanwenjunibv@163.com (W.Y.); bazhongsongzhou@163.com (Z.S.); lihaoibv@163.com (H.L.); 2017141241124@stu.scu.edu.cn (X.X.); gukui0404@stu.scu.edu.cn (K.G.); MA_peng99@163.com (P.M.); tymgood@126.com (Y.T.); l1z2l3y4@163.com (C.Z.); 2020322040035@stu.scu.edu.cn (Y.Z.); yangxin0822@163.com (X.Y.)

**Keywords:** infectious bronchitis virus, spike ectodomain, consensus nucleotide sequence, DNA vaccine, heterologous challenge

## Abstract

Avian coronavirus infectious bronchitis virus (IBV) causes severe economic losses in the poultry industry, but its control is hampered by the continuous emergence of new genotypes and the lack of cross-protection among different IBV genotypes. We designed a new immunogen based on a spike with the consensus nucleotide sequence (S_con) that may overcome the extraordinary genetic diversity of IBV. S_con was cloned into a pVAX1 vector to form a new IBV DNA vaccine, pV-S_con. pV-S_con could be correctly expressed in HD11 cells with corresponding post-translational modification, and induced a neutralizing antibody response to the Vero-cell-adapted IBV strain Beaudette (p65) in mice. To further evaluate its immunogenicity, specific-pathogen-free (SPF) chickens were immunized with the pV-S_con plasmid and compared with the control pVAX1 vector and the H120 vaccine. Detection of IBV-specific antibodies and cell cytokines (IL-4 and IFN-γ) indicated that vaccination with pV-S_con efficiently induced both humoral and cellular immune responses. After challenge with the heterologous strain M41, virus shedding and virus loading in tissues was significantly reduced both by pV-S_con and its homologous vaccine H120. Thus, pV-S_con is a promising vaccine candidate for IBV, and the consensus approach is an appealing method for vaccine design in viruses with high variability.

## 1. Introduction

Avian coronavirus infectious bronchitis virus (IBV), the type species of the *Gammacoronavirus* genus in the family *Coronaviridae* (https://talk.ictvonline.org/taxonomy/), is the pathogen causing infectious bronchitis (IB). IBV can infect chickens of all ages, affecting the trachea, kidney, oviduct, or gastrointestinal tract in a strain-dependent manner. Moreover, IBV infection causes severe economic losses in the poultry industry by reducing feed–gain ratio, egg production and quality, and even death [1,2]. IBV is an enveloped, non-segmented, mono-stranded, positive-sense RNA virus with a genome size of approximately 27.6 kb. Two-thirds of the IBV genome at the 5′-terminal encodes fifteen transcription-replication-associated non-structural proteins (NSP2–16), while the remaining genome encodes virion-assembly-related structural proteins (spike glycoprotein, small membrane protein, membrane glycoprotein, and phosphorylated nucleocapsid protein) and accessory proteins [1]. Among these proteins, the S glycoprotein is post-translationally cleaved into the amino-terminal S1 subunit and the carboxyl-terminal S2 subunit, the S1 subunit contains epitopes for neutralizing antibodies while the S2 subunit is also involved in inducing protective immunity [3,4,5].

IBV is associated with a high frequency of point mutations, insertions, deletions, and recombination [1,6]. Currently, IBV is comprised of seven genotypes that together include 36 distinct viral lineages and several unique variants [7,8,9]. Among these genotypes, GI-19 (also called QX-like) has expanded globally in recent years [2,6]. These different virus types do not cross-protect, making the control and prevention of IBV infection extremely difficult [10]. Live attenuated and killed vaccines developed from classical strains are most commonly used in IBV vaccination; however, they are usually not effective enough due to poor matching with field viruses [11].

A consensus approach that computationally designs vaccine immunogen sequences is widely used to overcome the extraordinary genetic diversity of RNA viruses. The resulting sequence consists of a nucleotide or amino acid that is most commonly used at each site, reducing the genetic distances to all original strains [12,13,14,15,16]. Previous studies have demonstrated that consensus-sequence-based vaccines elicit broader immune responses in both human immunodeficiency virus-1 (HIV-1) and influenza viruses compared with vaccines using naturally occurring sequences [15,17,18,19,20].

In this study, we constructed an IBV DNA vaccine containing the spike ectodomain with consensus nucleotide sequences and evaluated its immunogenicity.

## 2. Materials and Methods

### 2.1. Ethics Statement

All animal experiments in this study were approved by the Animal Ethics Committee (AEC) of Sichuan University (license: SYXK-Chuan-2018-185). All experimental procedures and animal welfare standards strictly followed the animal management guidelines of Sichuan University.

### 2.2. Viruses and Cells

IBV strains Beaudette (p65) and M41 were stored at −80 °C in our laboratory. Propagation and calculation of the 50% chicken embryo infectious dose (EID50) for M41 were performed as previously described [21,22]. The number of 10-day-old specific-pathogen-free (SPF) embryonated eggs (Boehringer Ingelheim Vital Biotechnology Co. Ltd., Beijing, China) used were 10 and 30, respectively. Cells (HD11 and Vero) were maintained in Dulbecco’s modified Eagle’s medium (DMEM) (HyClone, Logan, UT, USA) supplemented with 10% fetal bovine serum (FBS) (Gemini Bio-Products, West Sacramento, CA, USA) and 100 U penicillin-streptomycin (HyClone, Logan, UT, USA) [23]. Beaudette (p65) was amplified using Vero cells.

### 2.3. Design and Analysis of IBV S Protein Consensus Nucleotide Sequence

A total of 257 genome sequences of IBV strains isolated in China or used as a vaccine were collected from GenBank on 22 January 2018. These sequences were aligned using MAFFT v7.313 [24], and the consensus nucleotide sequence of spike (S-CON) was constructed by the Jalview program and listed in Supplementary Materials (Figures S1 and S2) [25]. Furthermore, the phylogenetic analysis of S-CON was carried out using MEGA (version 5.2.2).

### 2.4. Design and Construction of IBV DNA Vaccine

**Figure 1:**
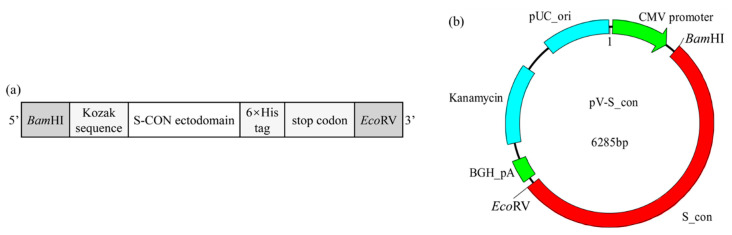
Schematic diagram of the S_con gene and infectious bronchitis virus (IBV) DNA vaccine (pV-S_con) plasmid. (**a**) The S_con gene. The Kozak sequence (GCCACCATGG) and 6 × His-Tag gene were added to ensure its correct expression in a eukaryotic expression vector and enable detection, respectively. (**b**) The pV-S_con plasmid. The S_con gene was inserted into the pVAX1 vector between the multiple cloning sites *Bam*HI and *Eco*RV.

To construct the IBV DNA vaccine, a eukaryotic expression gene box was designed (Figure 1a). Briefly, a restriction enzyme site *Bam*HI and a Kozak sequence (GCCACCATGG) were added at the 5′-terminus of the S-CON ectodomain (defined by TMHMM Server v. 2.0 (http://www.cbs.dtu.dk/services/TMHMM/)), and a 6 × His-Tag gene with two stop codons (TAATGA) and a restriction enzyme site *Eco*RV were fused to its 3′-terminus. The complete eukaryotic expression gene box was named S_con (the nucleotide sequence of S_con was listed in Supplementary Materials) and it was chemically synthesized by Sangon Biotech (Shanghai, China). The synthesized S_con was ligated with the pUC57 plasmid, and then cloned into the eukaryotic expression vector pVAX1, generating the target plasmid for the IBV DNA vaccine pV-S_con (Figure 1b).

### 2.5. Analysis of S_con Expression by Indirect Immunofluorescence Assay and Western Blot

Both the pVAX1 and pV-S_con plasmids were extracted from the host *E. coli* DH5α (TransGen Biotech, Beijing, China) by HiPure Plasmid EF Mini Kit (Magen, Guangzhou, China). The 80% to 85% confluent HD11 cells cultured in 6-well plates were transfected with 2 µg pVAX1 or pV-S_con with Lipofectamine 2000 (Invitrogen, CA, USA) in the ratio 1:2 (*w*/*v*). Forty-eight hours after transfection, cell expression was assessed by indirect immunofluorescence assay (IFA) and Western blot.

For the IFA, cells were washed twice with phosphate-buffered saline (PBS) (pH 7.2) before and after fixation with 4% paraformaldehyde for 20 min at room temperature. Cells were then sequentially incubated with QuickBlock™ Blocking Buffer for Immunol Staining (Beyotime, Shanghai, China) for 15 min at room temperature, 1:500 diluted mouse His-Tag Monoclonal Antibody (Proteintech, Rosemont, IL, USA) overnight at 4 °C, 1:80 diluted fluorescein (FITC)-conjugated Affinipure Goat Anti-Mouse IgG(H+L) (Proteintech, Rosemont, IL, USA) for 1 h at room temperature in the dark, and 4′,6-diamidino-2-phenylindole (DAPI) for 10 min at room temperature in the dark and washed thrice with PBS after each stage. Finally, cells were visualized using a Leica DMi8 microscope with Leica X software (Leica, Wetzlar, Germany) at × 100 magnification.

For the Western blot analysis, the cells were lysed and collected. After centrifugation of the cellular lysate, 10 μL of the supernatant was separated on a 10% SDS polyacrylamide gel. After electrophoresis, the proteins were transferred onto a PVDF membrane (Sangon Biotech, Shanghai, China), followed by incubation in 10% skim milk with TBST buffer for 1 h at room temperature. Next, the PVDF membrane was incubated with 1:10,000 diluted mouse His-Tag Monoclonal Antibody (Proteintech, Rosemont, IL, USA) overnight at 4 °C. The PVDF membrane was then washed 3 times in TBST and incubated with 1:5000 diluted HRP-conjugated Affinipure Goat Anti-Mouse IgG(H+L) (Proteintech, Rosemont, IL, USA) for 1 h at room temperature. Finally, after 3 washes in TBST, the protein was visualized using BeyoECL Plus kit (Beyotime, Shanghai, China) and imaged using Bio-Rad ChemiDoc Touch and its automatic chemiluminescence image analysis system (Bio-Rad, Irvine, CA, USA).

### 2.6. Neutralizing Antibody Induced by IBV S_con DNA Vaccine in Mice

Ten 5–6-week-old female BALB/c mice (Dossy Experimental Animals, Chengdu, China) were randomly divided into two groups. Two groups were immunized with 50 μg pVAX1 or pV-S_con through intramuscular injection. A booster immunization was performed 14 days later in the same manner at the same injection site. Fourteen days after the booster immunization, mice were sacrificed followed by blood collection. The serums were then separated and used for the virus neutralization (VN) assay. VN assays were performed by incubating each dilution of a 2-fold-diluted serum with 100TCID_50_ of the IBV Beaudette (p65) strain for 1 h at 37 °C. The incubating liquid was then used to infect the Vero cells. Neutralization titers were the highest dilution for which cells showed no cytopathic effect after 7 days of infection.

### 2.7. Immunization and Challenge

SPF White Leghorn chicken eggs (Boehringer Ingelheim Vital Biotechnology Co. Ltd., Beijing, China) were hatched by our laboratory. Thirty hatched chickens were fed until 7-days of age, and then randomly divided into three groups (i.e., pV-S_con, pVAX1, and H120). Chickens in the pV-S_con and pVAX1 groups were injected intramuscularly with 100 μg endotoxin-free pV-S_con and pVAX1 plasmids, respectively. Chickens in the H120 group were orally immunized with the H120 vaccine (Shandong Binzhou Wohua Bioengineering Co., Ltd., Shandong, China) according to the manufacturer’s instructions. On the 14th day post-primary vaccination (dpv), booster vaccinations were performed using the same protocol as the primary vaccination. On the 7th day after the booster vaccination, all chickens were challenged with 10^5.3^ EID_50_ IBV M41 (200 μL) via the nasal route.

### 2.8. Sample Collection

Serum samples of the chickens were collected through the veins under the wing on the 7th, 14th, 21st, and 28th dpv. All serum samples were used to measure IBV-specific antibody, while samples on the 14th and 28th dpv were also assessed for IL-4 and IFN-γ levels. Virus shedding after challenge was monitored with oral swabs on the 2nd, 5th, and 8th day post-challenge (dpc). After the challenge, all the chickens were monitored daily for clinical symptoms, and were sacrificed to obtain tissue samples (i.e., trachea, kidney, and cecal tonsil) at 10 dpc.

### 2.9. Evaluation the Efficacy of IBV S_con DNA Vaccine in Chickens

IBV-specific antibodies, IL-4, and IFN-γ were separately detected by the Infectious Bronchitis Virus Antibody Test Kit (IDEXX, Westbrook, ME, USA), ELISA Kit for Chicken Interleukin 4 (Jiangsu Meimian Industrial Co., Ltd., Jiangsu, China), and ELISA Kit for Chicken IFN-γ (Jiangsu Meimian Industrial Co., Ltd., Jiangsu, China).

IBV copies were detected using absolute quantification real-time PCR (qPCR) with RNAs extracted from oral swabs and tissue samples (i.e., trachea, kidney, and cecal tonsil). The primers (targeting the N gene of IBV strain M41) were 5′-GGTATAGTGTGGGTTGCTG CT-3′ (forward) and 5′-CTGCTGTTGATCTTCCACTCCT-3′ (reverse). All oral swabs were suspended in 500 µL PBS while 0.1 g of each tissue sample was mixed with 1 mL PBS and homogenized with a high-throughput tissue grinder (DHS life science, Beijing, China). RNAs were extracted as described by Guo et al. [26] using RNAiso Plus (Takara, Beijing, China) followed by genomic DNA removal and reverse transcription using PrimeScript™ RT reagent Kit with gDNA Eraser (Takara, Beijing, China). Finally, 2 µL cDNA (i.e., one-tenth of the total cDNA) was mixed with 10 µL of ChamQ Universal SYBR qPCR Master Mix (Vazyme Biotech Co., Ltd., Nangjing, China), 0.4 µL of each primer (10 µM), and 7.2 µL of sterile ddH_2_O to perform qPCR using Bio-Rad CFX connect (Bio-Rad, Carlsbad, CA, USA). The reaction protocol consisted of an initial denaturation for 5 min at 95 °C, followed by 40 cycles of 10 s at 95 °C, 30 s at 60 °C, and a melt curve at the end of all cycles. The pEASY-Blunt plasmid with IBV M41 N gene insertion was used to draw the standard curve, and all the qPCRs were repeated three times.

### 2.10. Statistical Analysis

Data analysis and graphical presentations were carried out using GraphPad Prism (v8.0.2) (GraphPad Software Inc., CA, USA). Significant difference (*p* < 0.05) and very significant difference (*p* < 0.01) were labelled as * and **, respectively.

## 3. Results

### 3.1. Phylogenetic Analysis of S-con

**Figure 2:**
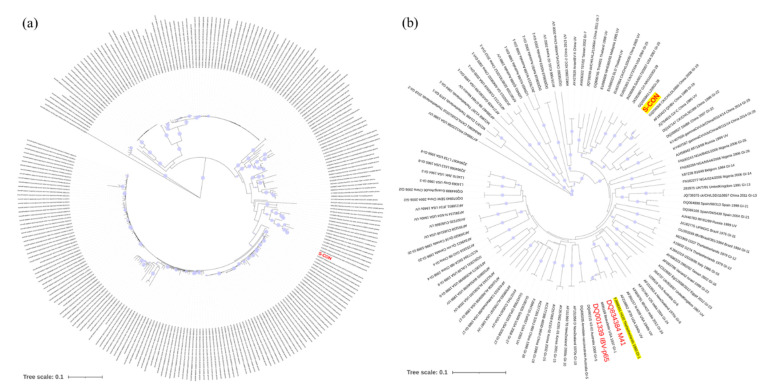
Phylogenetic analysis of the consensus nucleotide sequence of spike protein (S-CON). (**a**) Genetic relatedness of S-CON and indicator strains. A neighbor-joining (NJ) tree illustrated the genetic distances between the S-CON (red) nucleotide sequence and the indicator strains. (**b**) Genotypes of S-CON and strains used in this study. Bootstrap values (*n* = 1000 replicates) <70% were not shown.

Based on the nucleotide sequences of S-CON and the 257 strains used for consensus sequence analysis, S-CON was at the center of the phylogenetic tree (Figure 2a). In addition, S-CON and the IBV strains Beaudette (p65), M41, and H120 were genotyped according to a previous report [9]. S-CON was clustered in GI-19 while Beaudette (p65), M41, and the H120 grouping was in GI-1 (Figure 2b).

### 3.2. Analyses of S_con Protein Expression in Vitro

**Figure 3:**
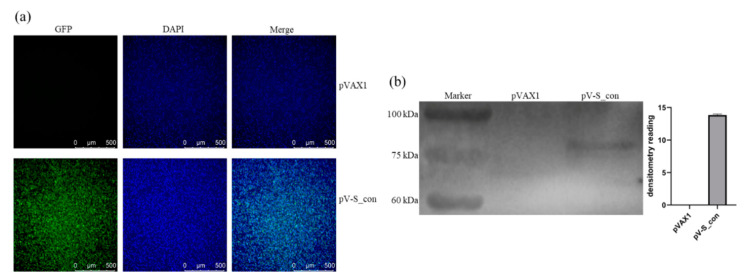
Expression of the S_con protein in vitro. (**a**) Expression of the S_con protein in HD11 cells analyzed by IFA. (**b**) Expression of the S_con protein in HD11 cells analyzed by Western blot.

The IBV S_con plasmid was transfected into HD11 cells, and the expression of the S_con protein was analyzed by IFA and Western blot. As shown in Figure 3a, the positive signal revealed in pV-S_con transfected cells but not in cells transfected with the control vector (pVAX1). Moreover, the lysates from cells transfected with pV-S_con or pVAX1 were assessed by Western blot. Only a single band (approximately 80 kDa) was detected in the lysates from cells transfected with pV-S_con (Figure 3b and Figure S2). The cleavage site of the furin protease at the boundary of subunits S1 and S2 was kept in S_con, so the approximately 80 kDa band in the Western blot analysis was the S2 subunit of the S_con protein. Taken together, the results indicate that the IBV S_con DNA vaccine plasmid could be successfully expressed and post-translationally modified in HD11 cells.

### 3.3. Neutralizing Antibody Induced by IBV S_con DNA Vaccine in Mice

**Figure 4:**
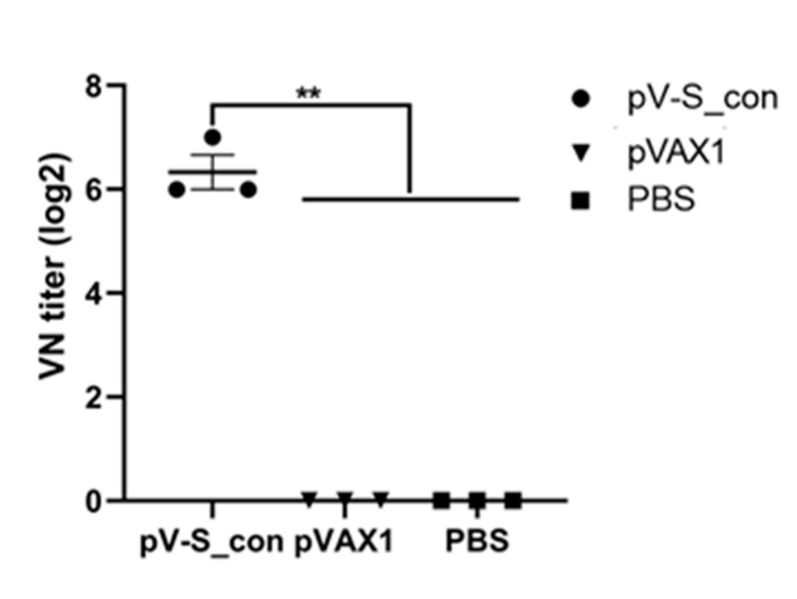
Neutralization titer of immunized mice against the IBV Beaudette (p65) strain. Serum of mice immunized with pV-S_con or pVAX1 were serially diluted twofold with PBS in microplates and mixed with 100 EID_50_ (50% chicken embryo infectious dose) of Beaudette (p65). PBS was used as the negative control. Virus neutralization (VN) titers were defined as the highest dilution where cells showed no cytopathic effect after 7 days of infection. There were three samples in each group and the value is expressed as the mean ± SEM. Ordinary one-way ANOVA was used for statistical analysis; ** *p* < 0.01.

The VN assay demonstrated that the serum of mice immunized with pV-S_con could neutralize Beaudette (p65) at a titer of 6.3 log2 while PBS and the serum of mice immunized with pVAX1 lacked neutralizing ability (Figure 4). This result indicates that the IBV S_con DNA vaccine is worthy of further study in chickens.

### 3.4. IBV-Specific Antibodies in Immunized Chickens

**Figure 5:**
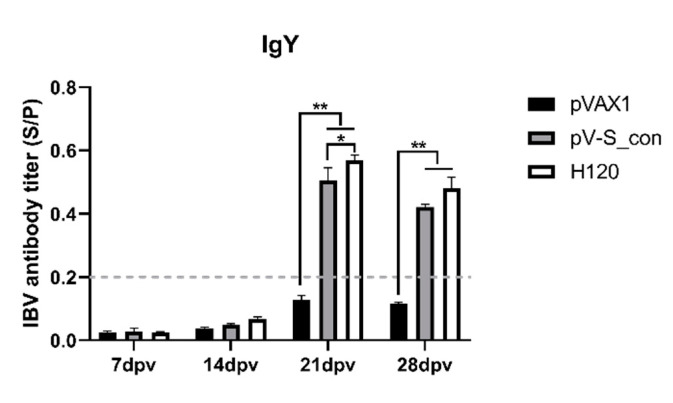
IBV-specific antibodies in the serum. The antibody titers are shown as mean sample vs. positive control (S/P) values + SEM of each group. The threshold cut-off values of the IBV ELISA were 0.2. S/P = (Sample A (650) − NC (negative control) A (650))/(PC (positive control) A (650) − NC A (650)). Tukey’s multiple comparison test was used for statistical analysis; * *p* < 0.05, ** *p* < 0.01.

On the 7th, 14th, 21st, and 28th dpv, IBV-specific antibodies in serum were evaluated by indirect ELISAs. On the 7th and 14th dpv, antibodies of all groups were seronegative (S/P < 0.2). Antibodies of the pV-S_con and H120 groups were seroconverted to positivity after the booster vaccination. As shown in Figure 5, the pV-S_con and H120 groups revealed very significantly (*p* < 0.01) higher IBV-specific antibody levels compared to the pVAX1 group at both the 21st and 28th dpv. Antibodies of groups pV-S_con and H120 reached the highest level on the 21st dpv, and the antibody levels of the H120 group were individually significantly higher (*p* < 0.05) and higher than the pV-S_con group at the 21st and 28th dpv.

### 3.5. Cytokines after Immunization in Chickens

**Figure 6:**
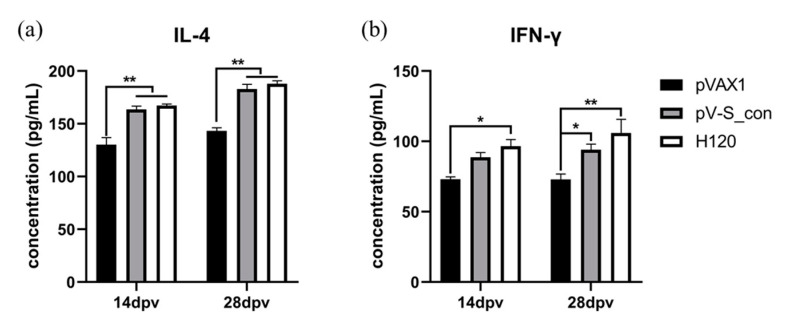
Analysis of the IL-4 (**a**) and IFN-γ (**b**) concentrations in the serum on the 14th and 28th dpv. Tukey’s multiple comparison test was used for statistical analysis. Concentrations are shown as mean values + SEM of each group; * *p* < 0.05, ** *p* < 0.01.

The cytokines IL-4 and IFN-γ were measured on the 14th and 28th dpv to assess the ability of the IBV S_con DNA vaccine to induce a cellular immune response. At both the 14th and 28th dpv, the IL-4 concentration of the pV-S_con group and group H120 were comparable, and both were very significantly higher than group pVAX1 (Figure 6a). The IFN-γ concentrations of the H120 group were significantly higher than the pVAX1 group and higher than the pV-S_con group at both the 14th and 28th dpv (Figure 6b). Moreover, the IFN-γ levels of the pV-S_con group were higher and significantly higher than the pVAX1 group on the 14th and 28th dpv, respectively.

### 3.6. Evaluation of Protection Against IBV M41 Challenge

**Figure 7:**
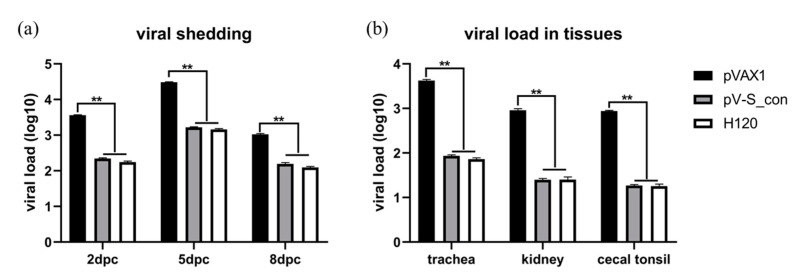
Viral shedding (**a**) and viral load in tissues (**b**) after M41 challenge. The formula of the standard curve is y = 37.302 − 3.155x, E = 107.5%, R^2^ = 0.988. A multiple *t*-test was used for statistical analysis. Viral loads are shown as mean values + SEM of each group; ** *p* < 0.01.

In this study, chickens were challenged with the virulent IBV strain M41 and kept for 10 days. After the challenge, the chickens in the pVAX1 group presented with mild cough and asthma while chickens in the pV-S_con and H120 groups had no apparent symptoms. To monitor the virus shedding, oral swabs were collected from the chickens on the 2nd, 5th, and 8th dpc for viral RNA detection. As shown in Figure 7a, the immunized groups (pV-S_con and H120) had very significantly reduced IBV RNA in the swabs after the challenge. At the 10th dpc, all the chickens were sacrificed and the tissues (i.e., trachea, kidney, and cecal tonsil) were collected and analyzed for IBV RNA. As shown in Figure 7b, the immunized groups (pV-S_con and H120) had very significantly reduced IBV RNA in the tissues. In both the analysis of the virus shedding and viral load in the tissues, the degree of reduced IBV RNA was not significantly different between the pV-S_con and H120 groups.

## 4. Discussion

IBV has circulated for nearly 90 years since it was first reported in 1931. Vaccination is considered the most cost-effective approach to controlling its infection [11]. Currently, the most commonly used IBV vaccines—live attenuated, or killed vaccines derived from classical or variant serotypes—can only act against strains of the same serotype [11]. Furthermore, the continuous emergence of new IBV serotypes makes the control and prevention of the virus infection more complex [6,27,28,29]. Moreover, the high cost and labor-intensive process hamper the development of vaccines against the new IBV variants. There is also the potential issue that the use of multiple strains of live vaccines may facilitate the formation of variants of viruses by recombination with field strains [30,31]. A vaccine based on consensus nucleotide sequences of type 2 PRRSV showed significantly broader levels of heterologous protection than wild-type PRRSV [16]. In this study, we aimed to develop a novel IBV vaccine based on spike with a consensus nucleotide sequence.

In this report, S-CON was constructed from 257 IBV strains isolated in China or used as vaccine. Consistent with the results of previous studies which found that GI-19 had become the predominant isolated type in China [32,33], our results showed that S-CON was grouped in GI-19 (Figure 2b). The S1 subunit of spike was found to mediate viral attachment to host cells and induce virus-neutralizing antibodies [34,35,36], and vaccines using S1 as antigen usually showed good protection against a homologous strain challenge [37,38,39]. However, recent work suggested that the S2 subunit of spike also contributed to protective immunity [3,4]. With this in mind, the ectodomain of the consensus spike was used as the immunogen in our study and was correctly expressed in HD11 cells (Figure 3) with corresponding post-translational modification (i.e., N-linked glycosylation and furin cleavage) and expression in other cells [37,40].

The IBV S_con DNA vaccine (pV-S_con) was injected into mice, and the serum obtained after immunization could efficiently neutralize Vero-cell-adapted IBV strain Beaudette (p65). Moreover, the VN titer was comparable to the titer of another IBV DNA vaccine (pV-S1B) after immunization of a chicken [41]. To further study its efficiency in inducing immunity, pV-S_con was inoculated in chickens, and the antibodies and cytokines in the serum were analyzed. The pV-S_con and H120 groups induced significantly higher IBV-specific antibody than group pVAX1 after the booster immunization (Figure 5). In agreement with our results, Belkasmi et al. and Okino et al. also revealed that antibodies were seronegative more than two weeks after immunization [42,43]. Many factors can influence the outcome of vaccination, and this is reviewed by de Wit and Cook [44]. The antibody titer of the pV-S_con group was lower than the H120 group, possibly because the coating antigen of the ELISA is whole IBV virions. In terms of the cellular immune responses, the pV-S_con group induced similar levels of IL-4 and IFN-γ compared to group H120 but had significantly higher levels than group pVAX1 (Figure 6). These results, demonstrate the ability of the pV-S_con vaccine to evoke both Th1- and Th2-type cellular immune responses [45].

A highly efficacious vaccine should be able to protect against virus challenge and prevent pathogen transmission. Our data show that the pV-S_con group suppressed the shedding and replication of heterologous IBV strain M41 as efficiently as its homologous vaccine H120 (Figure 7). In line with our results, vaccines based on the consensus sequence of the HIV-1 envelope and influenza virus hemagglutinin and neuraminidase showed protective capability against heterologous strains [15,16,17,18,19]. Our study only used the IBV GI-1 strain in the virus challenge, however, more strains in different genotypes should be tested in the future.

In conclusion, we designed a new IBV immunogen based on a spike with the consensus nucleotide sequence which was cloned into the pVAX1 vector to form a DNA vaccine (pV-S_con). Moreover, the novel DNA vaccine effectively protected chickens against a heterologous strain challenge. Our study confirmed that vaccines derived from spike with the consensus nucleotide sequence are a promising strategy for IBV vaccine development, and for other viruses with high variability.

## Data Availability

The data presented in this study are available on request from the corresponding author.

## Supplementary Materials

The following are available online at https://www.mdpi.com/2076-393X/9/1/50/s1. Figure S1: The amino acid sequence difference between S-CON, consensus S amino acid sequence (from 257 isolates), and two IBV strains (Beaudette (p65) and M41). Figure S2: The whole blot of Figure 3b. Nucleotide sequences of S-CON and S_con.
