# Supplementary material for: Design and Characterization of a DNA Vaccine Based on Spike with Consensus Nucleotide Sequence against Infectious Bronchitis Virus
Source: Vaccines (Basel). 2021 Jan 14;9(1):50. doi: 10.3390/vaccines9010050 (PMC7830736; doi:10.3390/vaccines9010050)
Supplement: Supplementary file 1 [file vaccines-09-00050-s001.pdf]

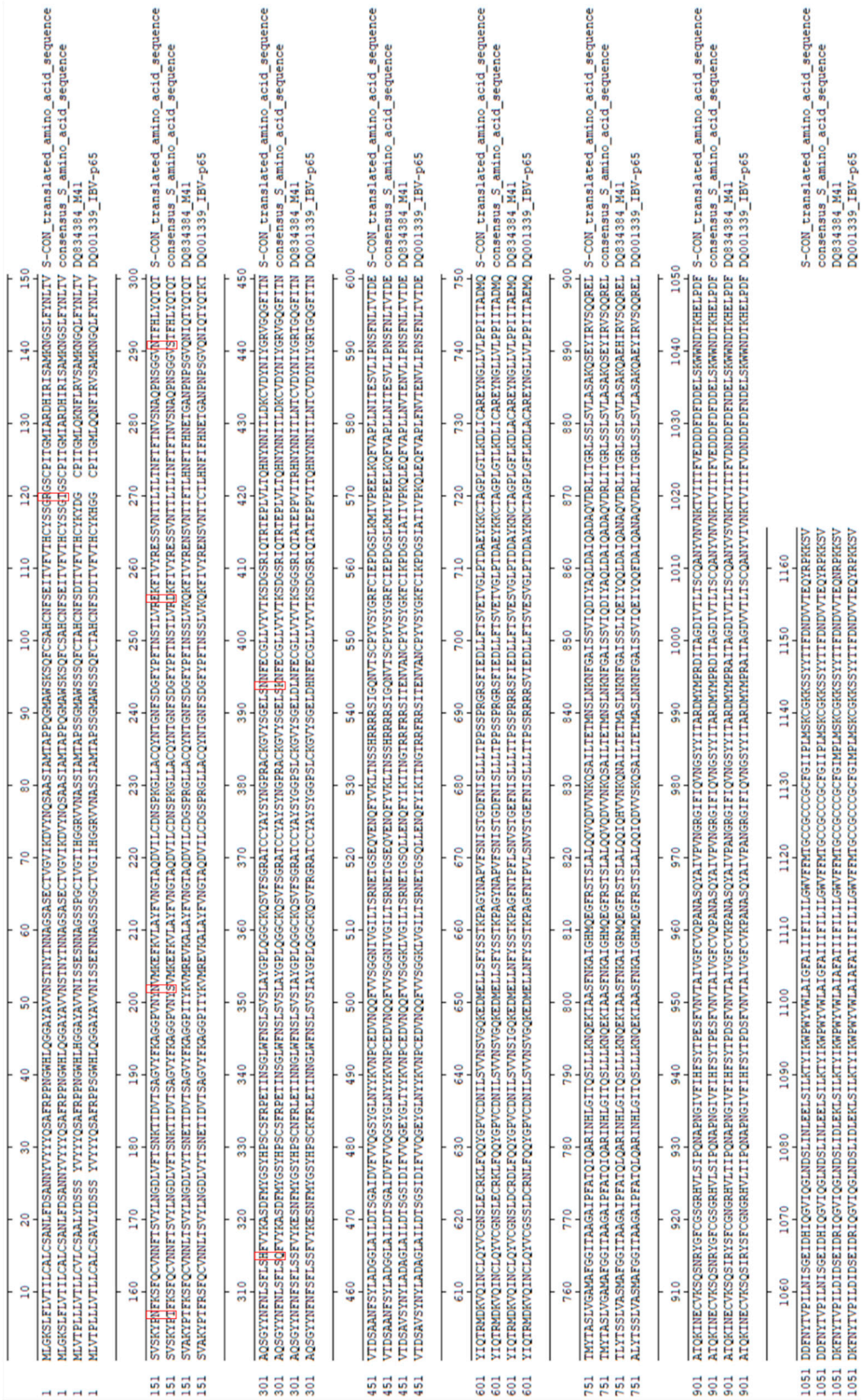

Figure S1. The amino acid sequence difference between S-CON, consensus S amino acid sequence (from 257 isolates), and two IBV strains (Beaudette (p65) and M41). S-CON translated amino acid sequence, consensus S amino acid sequence, Beaudette (p65) and M41 were aligned by MEGA (version 5.2.2). Different sites between S-CON and consensus S amino acid sequence were marked with red boxes.

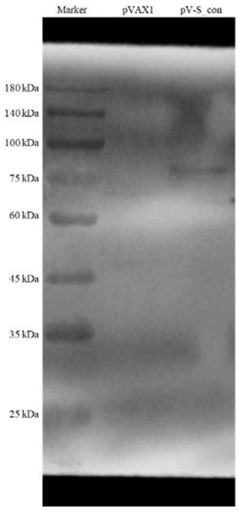


Figure S2. The whole blot of Figure 3b.

Nucleotide sequences of S-CON and S_con

>S-CON

ATGTTGGGGAAGTCACTGTTTTTAGTGACCATTTTGTGTGCACTATGTAGTGCAAATTT
GTTTGATTCTGCTAATAATTATGTGTACTACTACCAAAGTGCCTTTAGGCCTCCAAATGG
ATGGCATTTACAAGGGGGTGCTTATGCAGTAGTGAATTCTACTAATTATACTAATAATGC
AGGTTCTGCAAGTGAGTGCACTGTTGGTGTTATTAAGGACGTCTATAATCAAAGTGCGG
CTTCCATAGCTATGACAGCACCTCCTCAGGGTATGGCTTGGTCTAAGTCACAATTTTGTA
GTGCACACTGTAACTTTTCTGAAATTACAGTTTTTGTCACACATTGTTATAGTAGTGGTA
GAGGGTCTTGTCCTATAACAGGCATGATTGCACGTGATCATATTCGTATTTCTGCAATGA
AAAATGGTTCTTTATTTTATAATTTAACAGTTAGCGTATCTAAATACCCTAATTTTAAATC
TTTTCAATGTGTTAACAATTTCACATCTGTTTATTTAAATGGTGATCTTGTTTTTACTTCT
AATAAAACTACTGATGTTACGTCAGCAGGTGTGTATTTTAAAGCAGGTGGACCTGTAAA
TTATAATGTTATGAAAGAATTTAAGGTTCTTGCTTACTTTGTTAATGGTACAGCACAAGA
TGTAATTTTGTGTGATAATTCCCCTAAAGGTTTGCTAGCATGTCAATATAACACTGGCAA
TTTTTCAGATGGCTTTTATCCTTTTACTAATAGTACTTTAGTTAGGGAAAAGTTTATTGTC
TATCGTGAAAGTAGTGTTAATACTACTTTGACGTTAACTAATTTCACTTTTACTAATGTA
AGTAATGCACAGCCTAATAGTGGTGGTGTTAATACTTTTCATTTATATCAAACACAAACA
GCTCAGAGTGGTTATTATAATTTTAATTTGTCATTTCTGAGTCATTTTGTGTATAAGGCAA
GTGATTTTATGTATGGGTCTTACCACCCTAGTTGTTCTTTTAGACCAGAAACCATTAATA
GTGGTTTGTGGTTTAATTCATTGTCAGTTTCTCTTGCTTACGGACCCCTTCAAGGTGGG
TGTAAGCAATCTGTTTTTAGTGGTAGGGCAACGTGTTGTTATGCCTACTCTTATAATGGC
CCTAGGGCATGTAAAGGTGTTTATTCAGGTGAATTAAGCAATAATTTTGAATGTGGATTG
CTGGTTTATGTTACTAAGAGTGATGGCTCTCGTATACAAACTAGAACTGAGCCCTTAGT
ATTAACGCAACACAATTATAATAATATTACTTTAGATAAGTGTGTTGACTATAATATATATG
GCAGAGTTGGCCAAGGTTTTATTACTAATGTGACTGATTCTGCTGCTAATTTTAGTTATT
TAGCAGATGGTGGGTTAGCTATTTTAGATACTTCGGGTGCCATAGATGTCTTTGTTGTAC
AAGGCAGCTATGGTCTTAATTATTACAAGGTTAATCCTTGTGAAGATGTTAACCAACAG
TTTGTAGTGTCTGGTGGCAATATAGTTGGCATTCTTACTTCTAGAAATGAAACAGGTTCT
GAACAGGTTGAGAACCAGTTTTATGTTAAGTTAACCAATAGCTCACATCGTCGTAGGCG

TTCTATTGGCCAAAATGTAACAAGTTGTCCTTATGTTAGTTATGGCAGATTTTGTATTGA
ACCAGATGGTTCGTTAAAGATGATAGTGCCAGAAGAATTGAAACAGTTTGTGGCACCT
TTACTTAATATTACTGAAAGTGTACTCATACCTAACAGTTTTAATTTGACTGTTACAGAT
GAGTACATACAAACACGTATGGATAAGGTCCAAATCAATTGCCTTCAATATGTTTGCGG
CAATTCTTTGGAGTGTAGAAAATTGTTTCAACAATATGGTCCGGTTTGTGATAACATATT
GTCTGTTGTAAATAGTGTTGGTCAAAAAGAAGATATGGAACTTTTAAGTTTCTATTCTTC
TACTAAACCAGCTGGTTATAATGCACCTGTTTTTAGTAATATTAGTACTGGTGATTTTAAT
ATTTCTCTTTTATTAACACCACCTAGTAGTCCTAGAGGTCGTTCTTTTATAGAAGATTTGT
TATTTACAAGTGTTGAAACAGTTGGTTTGCCAACTGATGCTGAATATAAAAAATGCACA
GCAGGACCTTTAGGTACTCTTAAGGATCTTATCTGTGCTAGGGAATATAATGGTTTATTA
GTGTTGCCTCCAATTATTACGGCGGATATGCAAACAATGTATACTGCTTCTTTAGTTGGT
GCCATGGCTTTTGGTGGTATTACAGCAGCAGGTGCCATACCATTTGCAACTCAGATACA
AGCCAGAATTAATCATTTGGGTATTACACAGTCTCTGTTATTGAAAAATCAAGAGAAAA
TTGCAGCTTCCTTTAATAAGGCCATTGGTCATATGCAGGAAGGTTTTAGAAGCACTTCG
CTAGCATTACAACAGGTTCAAGACGTTGTTAATAAGCAGAGTGCTATTCTTACTGAAAC
TATGAATTCTCTTAATAAGAATTTTGGTGCTATTTCATCAGTAATTCAAGACATTTATGCG
CAACTTGATGCAATTCAAGCAGATGCACAAGTTGACCGTCTTATTACTGGCAGACTCTC
TTCTCTCTCAGTGCTAGCCTCTGCTAAGCAGTCTGAGTACATCAGAGTTTCCCAACAAC
GTGAATTAGCCACTCAAAAAATTAATGAGTGTGTTAAATCACAATCTAATAGGTATGGT
TTTTGTGGTAGTGGAAGACATGTTCTTTCGATACCACAAAATGCACCTAATGGTATAGT
GTTTATACACTTTAGTTACACTCCAGAGAGTTTTGTTAATGTTACTGCAATAGTAGGTTT
TTGTGTACAGCCTGCTAATGCTAGTCAGTATGCAATAGTGCCTGTTAATGGTAGAGGTAT
TTTTATACAAGTTAATGGTAGTTACTATATCACTGCACGTGATATGTATATGCCTAGAGAC
ATTACTGCAGGAGATATAGTTACTCTTACTTCTTGTCAAGCAAATTATGTTAATGTAAAT
AAAACCGTCATTACTACATTTGTGGAAGATGACGATTTTGATTTTGATGATGAGTTGTC
GAAATGGTGGAATGATACTAAGCATGAGCTACCAGACTTTGACGACTTCAATTATACAG
TACCCATACTTAATATTAGTGGTGAAATTGATCATATTCAAGGTGTTATACAGGGTCTTA
ATGACTCCCTTATAAACCTTGAAGAACTTTCAATACTTAAAACTTATATTAAGTGGCCTT
GGTATGTTTGGCTAGCCATAGGCTTTGCTATTATTATTTTTATCCTTATTTTAGGATGGGTG
TTTTTCATGACAGGTTGTTGTGGTTGTTGTTGTGGATGCTTTGGTATCATTCCTCTAATG
AGTAAGTGTGGTAAGAAATCTTCTTATTACACGACTTTTGATAATGATGTGGTAACTGA
ACAATACAGACCTAAAAAGTCTGTTTAA
>S_con
GGATCCGCCACCATGGGGAAGTCACTGTTTTTAGTGACCATTTTGTGTGCACTATGTAG
TGCAAATTTGTTTGATTCTGCTAATAATTATGTGTACTACTACCAAAGTGCCTTTAGGCC
TCCAAATGGATGGCATTTACAAGGGGGTGCTTATGCAGTAGTGAATTCTACTAATTATAC
TAATAATGCAGGTTCTGCAAGTGAGTGCACTGTTGGTGTTATTAAGGACGTCTATAATC
AAAGTGCGGCTTCCATAGCTATGACAGCACCTCCTCAGGGTATGGCTTGGTCTAAGTCA
CAATTTTGTAGTGCACACTGTAACTTTTCTGAAATTACAGTTTTTGTCACACATTGTTAT
AGTAGTGGTAGAGGGTCTTGTCCTATAACAGGCATGATTGCACGTGATCATATTCGTATT
TCTGCAATGAAAAATGGTTCTTTATTTTATAATTTAACAGTTAGCGTATCTAAATACCCTA
ATTTTAAATCTTTTCAATGTGTTAACAATTTCACATCTGTTTATTTAAATGGTGATCTTGT
TTTTACTTCTAATAAAACTACTGATGTTACGTCAGCAGGTGTGTATTTTAAAGCAGGTGG
ACCTGTAAATTATAATGTTATGAAAGAATTTAAGGTTCTTGCTTACTTTGTTAATGGTAC

AGCACAAGATGTAATTTTGTGTGATAATTCCCCTAAAGGTTTGCTAGCATGTCAATATAA
CACTGGCAATTTTTCAGATGGCTTTTATCCTTTTACTAATAGTACTTTAGTTAGGGAAAA
GTTTATTGTCTATCGTGAAAGTAGTGTTAATACTACTTTGACGTTAACTAATTTCACTTTT
ACTAATGTAAGTAATGCACAGCCTAATAGTGGTGGTGTTAATACTTTTCATTTATATCAA
ACACAAACAGCTCAGAGTGGTTATTATAATTTTAATTTGTCATTTCTGAGTCATTTTGTG
TATAAGGCAAGTGATTTTATGTATGGGTCTTACCACCCTAGTTGTTCTTTTAGACCAGAA
ACCATTAATAGTGGTTTGTGGTTTAATTCATTGTCAGTTTCTCTTGCTTACGGACCCCTT
CAAGGTGGGTGTAAGCAATCTGTTTTTAGTGGTAGGGCAACGTGTTGTTATGCCTACTC
TTATAATGGCCCTAGGGCATGTAAAGGTGTTTATTCAGGTGAATTAAGCAATAATTTTGA
ATGTGGATTGCTGGTTTATGTTACTAAGAGTGATGGCTCTCGTATACAAACTAGAACTG
AGCCCTTAGTATTAACGCAACACAATTATAATAATATTACTTTAGATAAGTGTGTTGACT
ATAATATATATGGCAGAGTTGGCCAAGGTTTTATTACTAATGTGACTGATTCTGCTGCTA
ATTTTAGTTATTTAGCAGATGGTGGGTTAGCTATTTTAGATACTTCGGGTGCCATAGATGT
CTTTGTTGTACAAGGCAGCTATGGTCTTAATTATTACAAGGTTAATCCTTGTGAAGATGT
TAACCAACAGTTTGTAGTGTCTGGTGGCAATATAGTTGGCATTCTTACTTCTAGAAATG
AAACAGGTTCTGAACAGGTTGAGAACCAGTTTTATGTTAAGTTAACCAATAGCTCACAT
CGTCGTAGGCGTTCTATTGGCCAAAATGTAACAAGTTGTCCTTATGTTAGTTATGGCAG
ATTTTGTATTGAACCAGATGGTTCGTTAAAGATGATAGTGCCAGAAGAATTGAAACAGT
TTGTGGCACCTTTACTTAATATTACTGAAAGTGTACTCATACCTAACAGTTTTAATTTGA
CTGTTACAGATGAGTACATACAAACACGTATGGATAAGGTCCAAATCAATTGCCTTCAA
TATGTTTGCGGCAATTCTTTGGAGTGTAGAAAATTGTTTCAACAATATGGTCCGGTTTGT
GATAACATATTGTCTGTTGTAAATAGTGTTGGTCAAAAAGAAGATATGGAACTTTTAAG
TTTCTATTCTTCTACTAAACCAGCTGGTTATAATGCACCTGTTTTTAGTAATATTAGTACT
GGTGATTTTAATATTTCTCTTTTATTAACACCACCTAGTAGTCCTAGAGGTCGTTCTTTTA
TAGAAGATTTGTTATTTACAAGTGTTGAAACAGTTGGTTTGCCAACTGATGCTGAATAT
AAAAAATGCACAGCAGGACCTTTAGGTACTCTTAAGGATCTTATCTGTGCTAGGGAATA
TAATGGTTTATTAGTGTTGCCTCCAATTATTACGGCGGATATGCAAACAATGTATACTGCT
TCTTTAGTTGGTGCCATGGCTTTTGGTGGTATTACAGCAGCAGGTGCCATACCATTTGC
AACTCAGATACAAGCCAGAATTAATCATTTGGGTATTACACAGTCTCTGTTATTGAAAA
ATCAAGAGAAAATTGCAGCTTCCTTTAATAAGGCCATTGGTCATATGCAGGAAGGTTTT
AGAAGCACTTCGCTAGCATTACAACAGGTTCAAGACGTTGTTAATAAGCAGAGTGCTA
TTCTTACTGAAACTATGAATTCTCTTAATAAGAATTTTGGTGCTATTTCATCAGTAATTCA
AGACATTTATGCGCAACTTGATGCAATTCAAGCAGATGCACAAGTTGACCGTCTTATTA
CTGGCAGACTCTCTTCTCTCTCAGTGCTAGCCTCTGCTAAGCAGTCTGAGTACATCAGA
GTTTCCCAACAACGTGAATTAGCCACTCAAAAAATTAATGAGTGTGTTAAATCACAATC
TAATAGGTATGGTTTTTGTGGTAGTGGAAGACATGTTCTTTCGATACCACAAAATGCAC
CTAATGGTATAGTGTTTATACACTTTAGTTACACTCCAGAGAGTTTTGTTAATGTTACTG
CAATAGTAGGTTTTTGTGTACAGCCTGCTAATGCTAGTCAGTATGCAATAGTGCCTGTTA
ATGGTAGAGGTATTTTTATACAAGTTAATGGTAGTTACTATATCACTGCACGTGATATGTA
TATGCCTAGAGACATTACTGCAGGAGATATAGTTACTCTTACTTCTTGTCAAGCAAATTA
TGTTAATGTAAATAAAACCGTCATTACTACATTTGTGGAAGATGACGATTTTGATTTTGA
TGATGAGTTGTCGAAATGGTGGAATGATACTAAGCATGAGCTACCAGACTTTGACGACT
TCAATTATACAGTACCCATACTTAATATTAGTGGTGAAATTGATCATATTCAAGGTGTTAT
ACAGGGTCTTAATGACTCCCTTATAAACCTTGAAGAACTTTCAATACTTAAAACTTATAT

TAAGTGGCCTCATCATCATCATCATCACTAATGATATC
